# Risk of perioperative mortality and venous thromboembolism after total hip or knee arthroplasty with recent COVID-19 infection: an observational study from the Kaiser Permanente Northern California Database

**DOI:** 10.2340/17453674.2025.44481

**Published:** 2025-09-17

**Authors:** Aidan T MORRELL, Ryland P KAGAN, Mackenzie KELLY, Graham J DEKEYSER, Andrew L AVINS, Lusine X GIGOYAN, John S COX

**Affiliations:** 1 Department of Orthopedics and Rehabilitation, Oregon Health & Science University, Portland, OR; 2 Division of Research, The Permanente Medical Group, Pleasanton, CA; 3 Department of Orthopedic Surgery, The Permanente Medical Group, Walnut Creek, CA, USA

## Abstract

**Background and purpose:**

Limited data exist on venous thromboembolism (VTE) and mortality risk in patients undergoing primary total hip (THA) or knee arthroplasty (TKA) following recent COVID-19 infection. We aimed to evaluate whether the timing of COVID-19 infection affects postoperative VTE and mortality risk after THA or TKA.

**Methods:**

Adult Kaiser Permanente Northern California members undergoing elective THA or TKA from 2020–2022 were identified using internal procedure codes. 33,520 patients with or without SARS-CoV-2 within 6 months preoperatively were compared. Multivariate Poisson regression was used to calculate incidence rate ratios (RRs) adjusted for demographics, comorbidities, and Covid vaccination status. The primary outcome was 90-day VTE (deep venous thrombosis or pulmonary embolism). The secondary outcome was 90-day mortality.

**Results:**

Among patients with recent COVID-19, the 90-day VTE rate was 0.3%, and the mortality rate was 2.5%. Recent COVID-19 within 6 to 12 weeks preoperatively did not significantly increase 90-day VTE risk (RR 1.0, 95% confidence interval [CI] 0.38–2.8) but was associated with increased 90-day mortality risk (RR 3.1, CI 1.7–5.4).

**Conclusion:**

Recent COVID-19 infection did not significantly impact VTE risk after THA or TKA. However, infection within 6 to 12 weeks preoperatively was associated with increased 90-day mortality.

Coronavirus disease 2019 (COVID-19), caused by the SARS-CoV-2 virus, has significantly impacted healthcare systems worldwide, with over 1.2 million deaths in the United States as of January 2025 [1]. Despite widespread vaccination and booster programs, COVID-19 continues to circulate globally, posing unique challenges in surgical care [2]. Primary total hip and knee arthroplasty (THA and TKA) are among the most performed procedures in orthopedics, with well-documented benefits in improving pain, function, and quality of life [3]. However, perioperative complications such as venous thromboembolism (VTE) and mortality remain critical concerns [4,5].

The emergence of COVID-19 has introduced new variables into the perioperative landscape, with evidence suggesting that recent COVID-19 infection may influence the risk of adverse outcomes following surgery. Early studies highlighted the significant impact of COVID-19 on mortality in non-elective orthopedic conditions, such as hip fractures [6,7]. However, less is known about how recent COVID-19 infection affects outcomes in elective arthroplasty patients, where optimized preoperative planning and patient selection are standard practice. We aimed to evaluate the association between the timing of recent COVID-19 infection and the risk of VTE and mortality following primary elective THA and TKA using data from a large, multicenter database.

## Methods

### Study design and setting

A retrospective cohort study was performed using the Kaiser Permanente Northern California (KPNC) database. KPNC is an integrated health system with 21 medical centers providing comprehensive care to more than 30% of insured residents in Northern California. Membership is racially and ethnically diverse, and reflects the Northern California population, excluding those in the highest and lowest income brackets [8].

This study was reported according to STROBE guidelines.

### Participants/study subjects

Adults aged 18 years and older undergoing elective THA or TKA between January 26, 2020, and December 31, 2022, at Kaiser Permanente Northern California (KPNC) were included. The study period was chosen to include the first diagnosed case of COVID-19 in the database, until present day. Data was extracted from KPNC’s Epic^®^ Electronic Health Record (EHR) and associated research databases. To ensure full capture of exposure and clinical characteristics, we excluded patients who were not continuously enrolled in the health plan for 12 months before their surgeries.

### Description of exposure, treatment, or surgery

The primary definition of the exposure variable was a known COVID infection within 6 months prior to surgery. COVID infection was defined as either a positive PCR test result or a COVID diagnosis in the patient’s EHR. During the study period, a standardized institutional protocol was in place requiring preoperative COVID-19 screening, including symptom assessment and PCR testing for all patients undergoing elective surgery. Secondary definitions of exposure were COVID infection within 12 weeks and 6 weeks of the patient’s surgery. The index date was defined as the patient’s date of surgery. The comparison group consisted of KPNC members from the same cohort who did not have a COVID infection within the defined exposure period prior to surgery.

### Aftercare

Standard practice within KPNC for patients undergoing elective total hip or knee arthroplasty is to provide post-event antithrombotic therapy, typically twice-daily 81 mg aspirin, for 30 days postoperatively for standard risk patients, and a direct oral anticoagulant (DOAC) for high-risk patients.

### Variables, outcome measures, data sources, and bias

The primary outcome was the occurrence of a VTE event (inpatient or outpatient) within 90 days following the index date. The secondary outcome was all-cause mortality within 90 days of the index date. We defined the VTE outcome using ICD codes for a deep venous thrombus (DVT) or a pulmonary embolus (PE). Within KPNC, typically a DVT is diagnosed by Doppler ultrasound exam, and a PE is diagnosed with a chest computerized tomography scan.

Patient mortality data was obtained from validated sources, including deaths recorded within the hospital system, state mortality files, and the U.S. National Death Index.

Sociodemographic and clinical variables included age, sex, self-reported race, Neighborhood Deprivation Index (NDI), body-mass index (BMI), and COVID vaccination status. The NDI is a validated composite variable ranging from –5 to +5 with more positive values indicating increasing neighborhood deprivation (e.g., poverty and unemployment) [9]. NDI quartiles were calculated using the full KPNC population. BMI was determined from the closest measurement to the surgical date that was within 365 days of the index date and categorized into 4 groups using the Centers for Disease Control and Prevention’s established cutoffs: < 18 for underweight, 18 to < 25 for healthy weight, 25 to < 30 for overweight, and ≥ 30 for obese [10].

COVID vaccination status was defined as follows: individuals were considered “fully vaccinated” if they had received both doses of either Pfizer or Moderna vaccines or 1 dose of the Janssen COVID vaccine within 12 months prior to the date of surgery; members who had received only 1 dose of either the Pfizer or Moderna vaccine within the same 12-month period were categorized as “partially vaccinated”, while individuals who did not meet these criteria were classified as “unvaccinated”. The numbers of partially vaccinated patients were so small that these individuals were grouped with the unvaccinated group.

### Statistics

Descriptive statistics were reported for assessing the distribution of sociodemographic and clinical characteristics within both the exposed and unexposed cohorts. Continuous variables were summarized with medians and interquartile ranges (IQRs), while categorical variables were summarized with frequencies and percentages. Risk ratios (RR) were used to evaluate the adjusted and unadjusted risks of mortality and VTE within our cohort. Given the small sample sizes, we calculated Fisher’s exact tests for the relevant hypothesis tests. Multivariate adjustment was conducted with Poisson regression models, using robust standard errors. All statistical analyses were conducted using R statistical software, version 4.2.1 (R Foundation for Clinical Computing, Vienna, Austria), and all tests were two-sided, with the alpha level set at 0.05.

### Ethics, funding, and potential conflicts of interest

This study was conducted under approval by the Kaiser Permanente Northern California Institutional Review Board (approval #2056765). Data sharing is not possible due to existing regulation; however, requests for sub-analyses may be considered. This study was funded internally through the Kaiser Delivery Science and Applied Research Unit. Study funding did not influence the interpretation of data, reporting of results, writing of the manuscript, or decision to publish. Each author certifies that he or she has no commercial associations that might pose a conflict of interest in connection with the submitted article. Complete disclosure of interest forms according to ICMJE are available on the article page: 10.2340/17453674.2025.44481

## Results

The study cohort was derived from 45,661 Kaiser Permanente Northern California (KPNC) patients who underwent major orthopedic surgery between February 2020 and December 2022 (Figure). After excluding 80 patients younger than 18 years, 45,581 adult patients remained. Of these, 8,387 patients were excluded due to lack of continuous KPNC membership, leaving 37,194 eligible patients. An additional 3,674 patients who did not undergo hip or knee arthroplasty were excluded. The final analytic cohort consisted of 33,520 patients who underwent hip or knee arthroplasty.

**Figure:**
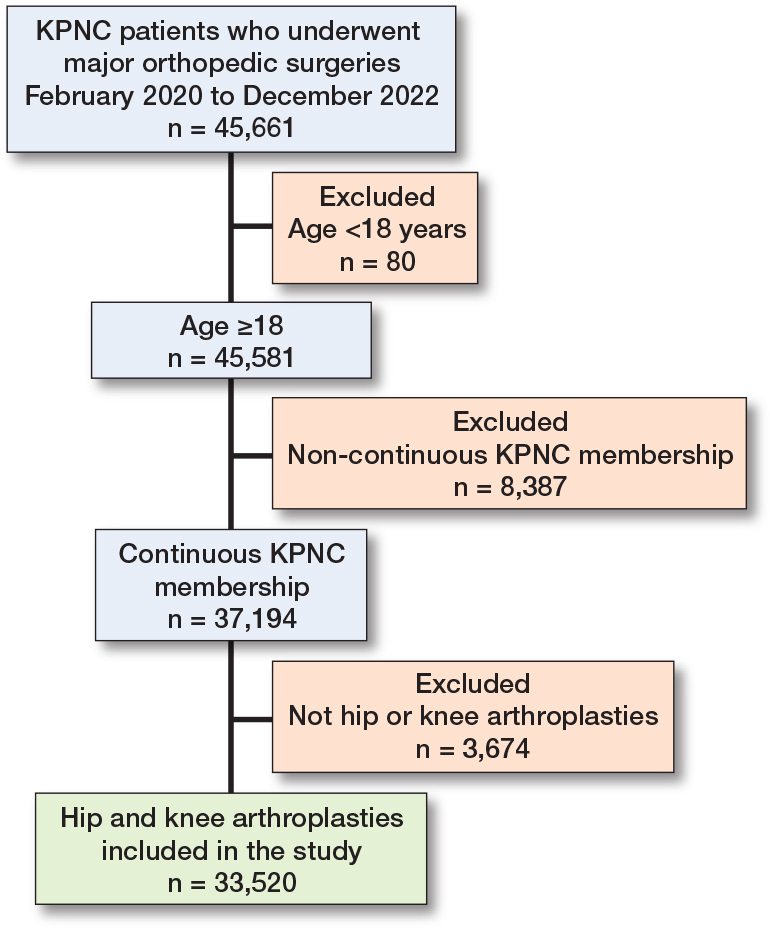
Patient study flow diagram.

Table 1 presents the distribution of baseline sociodemographic and clinical characteristics by exposure group. Most patients were older (> 65 years of age), female, and white. The great majority of patients (92.7%) were fully vaccinated against COVID prior to their surgeries. 474 patients died; causes of death are included in Supplementary data.

**Table 1:** Sociodemographics, clinical characteristics, and outcomes of patients who underwent total hip or knee replacement by preoperative COVID vaccination status. Values are count (%)

Characteristic	Total n = 33,520	Fully vaccinated n = 31,085	Unvaccinated n = 2,435 ^a^
Age at surgery			
< 55	2,481 (7.4)	2,169 (7.0)	312 (13)
56–65	8,500 (25)	7,728 (25)	772 (32)
> 65	22,539 (67)	21,188 (68)	1,351 (55)
Biological sex			
Female	19,611 (59)	18,315 (59)	1,296 (53)
Male	13,909 (41)	12,770 (41)	1,139 (47)
Race/ethnicity			
White	24,025 (72)	22,181 (71)	1,844 (76)
Hispanic	4,214 (13)	3,919 (13)	295 (12)
Black	2,129 (6.4)	1,973 (6.3)	156 (6.4)
Asian	2,597 (7.7)	2,505 (8.1)	92 (3.8)
Other/unknown	555 (1.7)	507 (1.6)	48 (2.0)
Neighborhood Deprivation Index (quartiles)			
Q1 (low deprivation)	9,450 (28)	9,002 (29)	448 (18)
Q2	11,064 (33)	10,292 (33)	772 (32)
Q3	8,323 (25)	7,584 (24)	739 (30)
Q4 (high deprivation)	4,683 (14)	4,207 (14)	476 (20)
Baseline BMI			
< 18.5	504 (1.5)	417 (1.3)	87 (3.6)
18.5–25	6,655 (20)	6,136 (20)	519 (21)
26–30	11,326 (34)	10,580 (34)	746 (31)
≥ 30	15,031 (45)	13,949 (45)	1,082 (44)
Unknown	4 (< 1)	3 (< 1)	1 (< 1)
Surgery type			
Knee replacement	17,709 (53)	16,684 (54)	1,025 (42)
Hip replacement	15,811 (47)	14,401 (46)	1,410 (58)
COVID 6 months pre-op.			
No COVID	32,373 (97)	30,039 (97)	2,334 (96)
COVID	1,147 (3.4)	1,046 (3.4)	101 (4.1)
COVID 12 weeks pre-op.			
No COVID	32,967 (98)	30,575 (98)	2,392 (98)
COVID	553 (1.6)	510 (1.6)	43 (1.8)
COVID 6 weeks pre-op.			
No COVID	33,288 (99)	30,875 (99)	2,413 (99)
COVID	232 (0.7)	210 (0.7)	22 (0.9)
VTE within 90 days of surgery			
No VTE	33,406 (100)	30,983 (100)	2,423 (100)
VTE	114 (0.3)	102 (0.3)	12 (0.5)
All-cause mortality within 90 days of surgery			
Alive	33,046 (99)	30,840 (99)	2,206 (91)
Died	474 (1.4)	245 (0.8)	229 (9.4)

a The numbers of partially vaccinated patients were so small that these individuals were grouped with the unvaccinated group.

### COVID-19 infection and the risk of postoperative VTE after primary THA or TKA

The overall incidence of VTE was 0.3% of all patients. Among those with a COVID-19 infection in the prior 6 months, 4 patients (0.4%) experienced a VTE event within 90 days of joint replacement surgery, with an unadjusted risk ratio (RR) of 1.0 (95% confidence interval [CI] 0.38–2.8; Table 2). In patients with a COVID-19 infection within 12 weeks of surgery, 2 VTE events (0.4%) were observed (RR 1.1, CI 0.26–4.3; Table 2). Additionally, there was no evidence of an association between VTE risk and COVID-19 infection within 6 weeks of surgery (RR 1.3, CI 0.18– 9.1; Table 2). Due to the very low number of observed VTE events, multivariable analyses were not feasible. Additional results separated by surgery type are available in the Supplementary data.

**Table 2:** Risk of venous thromboembolic event within 90 days of surgery for patients undergoing total knee arthroplasty or total hip arthroplasty with COVID infection within 6 months, 12 weeks, and 6 weeks of surgery (univariable analysis) compared with non-infected with the same follow-up

Follow-up or COVID infection at	COVID infection		No COVID infection		Rate ratio (CI)
	Total, n	Developed VTE, n (%)	Total, n	Developed VTE, n (%)	
6 months	1,147	4 (0.35)	32,373	110 (0.34)	1.0 (0.38–2.8)
12 weeks	553	2 (0.36)	32,967	112 (0.34)	1.1 (0.26–4.3)
6 weeks	232	1 (0.43)	33,288	113 (0.34)	1.3 (0.18–9.1)

### COVID-19 infection and the risk of mortality after primary THA or TKA

90-day postoperative mortality occurred in 474 patients (1.4%) across the total study cohort (Table 3). On univariable analysis, recent COVID-19 infection within 6 months of surgery was significantly associated with increased mortality risk (RR 1.8, CI 1.3–2.7; Table 3). However, after adjusting for baseline variables, the association was no longer statistically significant (RR 1.4, CI 0.93–2.0; Table 4). When the exposure window was restricted to COVID-19 infections within 12 weeks and 6 weeks prior to surgery, the association with postoperative mortality remained statistically significant in both univariable and multivariable analyses (RR 2.4, CI 1.5–3.8 and RR 3.1, CI 1.7–5.4, respectively; Tables 3 and 4). Significant predictors of 90-day postoperative mortality included older age, male sex, and lack of preoperative COVID-19 vaccination, while higher BMI was protective. Notably, across all 3 preoperative COVID-19 exposure windows (within 6 months, 12 weeks, and 6 weeks), no 90-day postoperative deaths were observed among fully vaccinated patients. This finding suggests a potentially protective effect of full vaccination against mortality in the setting of recent COVID-19 infection.

**Table 3:** Risk of all-cause mortality within 90 days of surgery for patients undergoing total knee arthroplasty or total hip arthroplasty by time with COVID infection within 6 months, 12 weeks, and 6 weeks of surgery (univariable analysis) compared with non-infected with the same follow-up

Follow-up or COVID infection at	COVID infection		No COVID infection		Rate ratio (CI)
	Total, n	Died, n (%)	Total, n	Died, n (%)	
6 months	1,147	29 (2.5)	32,373	445 (1.4)	1.8 (1.3–2.7)
12 weeks	553	19 (3.4)	32,967	455 (1.4)	2.5 (1.6–3.9)
6 weeks	232	16 (6.9)	33,288	458 (1.7)	5.0 (3.1– 8.1)

**Table 4:** Risk of all-cause mortality within 90 days of surgery for patients undergoing total knee arthroplasty or total hip arthroplasty by time with COVID infection within 6 months, 12 weeks, and 6 weeks of surgery (multivariable analysis) compared with non-infected with the same follow-up

Characteristic	Rate ratio (CI): multivariable analysis		
	6 months	12 weeks	6 weeks
COVID infection (ref. no COVID)			
COVID	1.40 (0.93–2.01)	2.37 (1.46–3.82)	3.05 (1.74–5.36)
COVID vaccination (ref. fully vaccinated)			
Unvaccinated	5.65 (4.65–6.86)	8.53 (7.00–10.4)	8.49 (6.97–10.4)
Age (ref. < 55)			
56–65	1.56 (0.52–6.69)	1.75 (0.53–5.73)	1.76 (0.54–5.77)
> 65	4.12 (1.56–16.7)	7.62 (2.50–23.2)	7.60 (2.50–23.2)
Sex (ref. female)			
Male	1.44 (1.19–1.75)	1.35 (1.12–1.62)	1.35 (1.12–1.62)
Race/ethnicity (ref. white)			
Hispanic	1.12 (0.80–1.52)	1.10 (0.82–1.48)	1.11 (0.83–1.49)
Black	1.02 (0.64–1.54)	1.00 (0.64–1.55)	1.00 (0.64–1.55)
Asian	1.02 (0.72–1.40)	1.05 (0.77–1.41)	1.05 (0.78–1.42)
Other/unknown	1.35 (0.53–2.77)	1.27 (0.64–2.55)	1.25 (0.63–2.50)
Neighborhood Deprivation Index (quartile) (ref. Q1 low deprivation)			
Q2	1.12 (0.88–1.44)	1.19 (0.94–1.50)	1.18 (0.94–1.50)
Q3	1.02 (0.78–1.32)	1.14 (0.88–1.46)	1.13 (0.88–1.46)
Q4 (high depr.)	1.01 (0.74–1.37)	1.17 (0.87–1.58)	1.17 (0.87–1.58)
Baseline BMI (ref. < 18.5)			
18.5–25	0.83 (0.63–1.12)	0.64 (0.48–0.85)	0.64 (0.48–0.86)
26–30	0.60 (0.43–0.84)	0.26 (0.18–0.37)	0.26 (0.18–0.37)
≥ 30	0.38 (0.25–0.57)	0.11 (0.08–0.17)	0.11 (0.08–0.17)

## Discussion

We aimed to evaluate the association between the timing of recent COVID-19 infection and the risk of VTE and mortality following primary elective THA and TKA . We found that recent COVID-19 infection did not significantly increase the risk of VTE in these patients. However, patients with a COVID-19 infection within 6 to 12 weeks prior to surgery demonstrated a higher risk of 90-day mortality compared with those without recent infection.

### COVID-19 infection and the risk of postoperative VTE after primary THA or TKA

We found no evidence of a difference in the unadjusted risk of VTE for patients with recent COVID-19 infection from 6 months to 6 weeks prior to total joint arthroplasty. These data suggest the risk of VTE may be lower than what has been reported previously, as Lee et al. noted a significantly higher rate of DVT among patients with COVID-19 infection up to 6 months prior to surgery using a national claims insurance database [11]. Of note, the analyses in that study were unable to account for vaccination status, which may be one reason for the difference in reported risk. Compared with a study using the Medicare Limited Data Set that did account for vaccination status, our findings still suggested the risk of VTE may still be lower. Okewunmi et al. observed that a COVID-19 infection before TKA was associated with increased odds of VTE; however, this association was not significant for THA or when PE and DVT were evaluated separately [12]. The analyses in this study were unable to account for timing of prior COVID-19 infection, which may be one reason for the difference in reported risk. In contrast to earlier studies that reported elevated venous thromboembolism (VTE) risk in COVID-19-positive arthroplasty patients, our findings did not demonstrate a significant difference in VTE incidence. A recent meta-analysis by Jackson et al. [13] summarized increased VTE risk across multiple studies [14-16]; however, most of these studies were based on data collected before or during 2021. As such, they may not reflect the evolving landscape of COVID-19 variants, widespread vaccination, or the implementation of standardized perioperative protocols. Our study, which includes data to the end of 2022, offers a more contemporary perspective. The lower observed VTE risk in our cohort may be attributable to several key factors: higher vaccination rates, more precise classification of infection timing, and consistent perioperative management within an integrated healthcare system. These distinctions likely contribute to the discrepancy between our findings and those of earlier studies.

### COVID-19 infection and the risk of mortality after primary THA or TKA

An increased risk of 90-day mortality was demonstrated if a COVID-19 infection occurred from 6 weeks to 12 weeks prior to surgery. It is difficult to compare our findings with other studies given the limited available literature, though a relevant difference was noted from the National COVID Cohort Collaborative who reported a loss of significance in the increased risk of 30-day mortality for THA or TKA for patients when they were diagnosed with COVID-19 ≥ 31 days before surgery [17]. Again, the analyses in that study were unable to account for vaccination status, which has been shown to affect perioperative complication and risk profiles and may account for the differences in our results [18]. An important and novel observation from our study is that no postoperative deaths occurred among fully vaccinated patients with recent COVID-19 infection, regardless of timing. This finding highlights the potential protective role of vaccination in mitigating severe postoperative outcomes, even in patients with recent infection. While our study was not powered to evaluate vaccine efficacy directly, this result supports the continued emphasis on vaccination as a critical component of preoperative risk reduction strategies.

In addition to infection timing and vaccination status, our adjusted models revealed that higher BMI (≥ 26) was associated with a reduced risk of 90-day mortality, a finding that aligns with the “obesity paradox” described in prior surgical literature [19]. Interestingly, subgroup analyses also showed reduced mortality risk among patients with normal BMI (18.5–25) in the 6- and 12-week exposure windows, particularly among older, unvaccinated males. These patterns suggest that BMI may either mediate the relationship between COVID-19 infection and mortality or exert an independent protective effect. Future research should explore this through mediation analysis and sensitivity testing using alternative BMI thresholds to better understand the robustness and implications of this association.

### Limitations

Most importantly, the retrospective design may introduce potential for bias in both exposure and outcome classification. Although we used a large, integrated healthcare system with standardized protocols and comprehensive data capture, retrospective analyses are inherently limited by unmeasured confounding and incomplete clinical detail. For example, we were unable to identify asymptomatic COVID-19 infections or stratify by infection severity. While a standardized institutional protocol required preoperative symptom screening and PCR testing, undiagnosed mild or asymptomatic infections may still have been missed. Vaccination status—potentially correlated with symptom burden—was associated with mortality outcomes, but we could not fully disentangle these effects. Additionally, VTE events were identified based on clinical documentation of DVT or PE in the electronic health record. As no universal screening was performed across all 33,520 cases, subclinical or unreported VTEs may have been missed, potentially leading to underestimation of the true incidence. We also could not account for all perioperative variables, such as differences in VTE prophylaxis regimens. These limitations are consistent with other large database studies and highlight the importance of future prospective research to validate and expand on these findings [20,21].

### Conclusions

Recent COVID-19 infection did not significantly impact VTE risk after THA or TKA. However, infection within 6 to 12 weeks preoperatively was associated with increased 90-day mortality.

*In perspective,* these results highlight the importance of carefully considering the timing of elective arthroplasty in patients with recent COVID-19 infection. While the risk of VTE appears unaffected, the increased mortality risk underscores the need for thorough preoperative evaluation and patient optimization. Policies mandating a fixed delay for elective surgery after a recent COVID-19 infection may still be premature. Further research is needed to clarify the underlying mechanisms of increased risk and identify strategies to mitigate adverse outcomes. Additionally, the absence of mortality among fully vaccinated patients with recent COVID-19 infection underscores the potential protective benefit of vaccination and supports its role in preoperative optimization. These findings may also inform perioperative planning in the context of other respiratory viral illnesses. For example, recent influenza infection, though less studied in the arthroplasty population, has similarly been associated with transient systemic inflammation and increased perioperative risk in other surgical cohorts. As seasonal influenza continues to circulate alongside SARS-CoV-2, future studies should evaluate whether similar timing-based risk profiles exist and whether vaccination confers comparable protective effects. Integrating lessons from COVID-19 may help refine surgical timing and risk stratification strategies for patients recovering from respiratory viral infections more broadly.

### Supplementary data

Supplementary Tables 1–3 are available as supplementary data on the article page, doi: 10.2340/17453674.2025.44481

## Supplementary Material



## Notes

ATM: conceptualization, analyzing and interpreting data, drafting and revising the manuscript. RK: conceptualization, analyzing and interpreting data, revising the manuscript. MK: interpreting data, revising the manuscript. GD: conceptualization, analyzing and interpreting data, revising the manuscript. AA: analyzing and interpreting data, revising the manuscript. LG: analyzing and interpreting data, revising the manuscript. JC: conceptualization, analyzing and interpreting data, drafting and revising the manuscript; guarantor of the work.

Handling co-editors: Per Henrik Randsborg

*Acta* thanks Per Kjærsgaard-Andersen, Anne Lübbeke, and Petri Virolainen for help with peer review of this manuscript.
