# Supplementary material for: Risk of perioperative mortality and venous thromboembolism after total hip or knee arthroplasty with recent COVID-19 infection: an observational study from the Kaiser Permanente Northern California Database
Source: Acta Orthop. 2025 Sep 17;96:692–7. doi: 10.2340/17453674.2025.44481 (PMC12444792; doi:10.2340/17453674.2025.44481)
Supplement: Supplementary file 1 [file ActaO-96-44481-s1.pdf]

Supplementary Data

Supplementary Table 1. Patients undergoing total knee arthroplasty; predictor is any COVID infection within 6 months, 12 weeks, and 6 weeks of surgery (univariable analysis)

	**Covid infection**		**No Covid infection**		
**Follow up or Covid within**	**Total, n**	**Developed VTE, n (%)**	**Total, n**	**Developed VTE, n (%)**	**Rate ratio (CI)**
6 months	605	2 (0.33)	17,104	42 (0.25)	1.35 (CI 0.33–5.55)
12 weeks	287	0 (0)	17,422	44 (0.25)	–
6 weeks	103	0 (0)	17,709	44 (0.25)	–

Supplementary Table 2. Patients undergoing total hip arthroplasty; predictor is any COVID infection within 6 months, 12 weeks, and 6 weeks of surgery (univariable analysis)

	**Covid infection**		**No Covid infection**		
**Follow up or Covid within**	**Total, n**	**Developed VTE, n (%)**	**Total, n**	**Developed VTE, n (%)**	**Rate ratio (CI)**
6 months	542	2 (0.37)	15,269	68 (0.45)	0.83 (CI 0.20–3.37)
12 weeks	266	2 (0.75)	15,545	68 (0.44)	1.72 (CI 0.42–6.98)
6 weeks	129	1 (0.78)	15,682	69 (0.44)	1.76 (CI 0.25–12.6)

Supplementary Table 3. Causes of death

Immediate/primary cause of death	n	%	Cumulative frequency
Acute myeloid leukemia	1	0.2	1
Acute myocardial infarction, unspecified	7	1.5	8
Acute renal failure, unspecified	2	0.4	10
Acute respiratory failure	34	7.2	44
Alzheimer's disease, unspecified	33	7.0	77
Anoxic brain damage, not elsewhere classified	1	0.2	78
Asphyxia	1	0.2	79
Atherosclerotic heart disease	4	0.8	83
Bladder, unspecified	3	0.6	86
Bradycardia, unspecified	1	0.2	87
Brain, unspecified	1	0.2	88
Breast, unspecified	4	0.8	92
Bronchus or lung, unspecified	10	2.1	102
COVID-19	6	1.3	108
Cachexia	7	1.5	115
Cardiac arrest, unspecified	76	16	191
Cardiogenic shock	2	0.4	193
Cerebral atherosclerosis	6	1.3	199
Cerebral edema	1	0.2	200
Cerebral infarction, unspecified	1	0.2	201
Chronic kidney disease, stage 5	8	1.7	209
Chronic myeloproliferative disease	1	0.2	210
Chronic obstructive pulmonary disease, unspecified	4	0.8	214
Colon, unspecified	2	0.4	216
Congestive heart failure	17	3.6	233

**Immediate/primary cause of death**	**n**	**%**	**Cumulative frequency**
Degenerative disease of nervous system, unspecified	4	0.8	237
Dependence syndrome	2	0.4	239
Dysphagia	2	0.4	241
Endocarditis, valve unspecified	1	0.2	242
Endometrium	1	0.2	243
Enterocolitis due to Clostridium difficile	1	0.2	244
Esophagus, unspecified	2	0.4	246
Essential (primary) hypertension	1	0.2	247
Fracture of femur, part unspecified	4	0.8	251
Fracture of neck of femur	14	3.0	265
Fracture of neck, part unspecified	1	0.2	266
Gastrointestinal hemorrhage, unspecified	6	1.3	272
Generalized and unspecified atherosclerosis	2	0.4	274
Heart failure, unspecified	5	1.1	279
Hypertensive heart disease without (congestive) heart failure	2	0.4	281
Hypovolemic shock	1	0.2	282
Intracerebral hemorrhage, unspecified	1	0.2	283
Intrahepatic bile duct carcinoma	2	0.4	285
Ischemic cardiomyopathy	1	0.2	286
Liver cell carcinoma	1	0.2	287
Malaise and fatigue	2	0.4	289
Malignant melanoma of skin, unspecified	1	0.2	290
Malignant neoplasm of kidney, except renal pelvis	1	0.2	291
Malignant neoplasm of ovary	2	0.4	293
Malignant neoplasm of parotid gland	1	0.2	294

Immediate/primary cause of death	n	%	Cumulative frequency
Malignant neoplasm of prostate	3	0.6	297
Malignant neoplasm of rectum	2	0.4	299
Malignant neoplasm without specification of site	4	0.8	303
Multiple fractures, unspecified	1	0.2	304
Multiple myeloma	4	0.8	308
Multiple sclerosis	1	0.2	309
Open wound of lip and oral cavity	1	0.2	310
Open wound of thorax, part unspecified	1	0.2	311
Other and unspecified abnormalities of breathing	2	0.4	313
Other and unspecified convulsions	1	0.2	314
Other and unspecified drugs, medicaments, and biological substances	2	0.4	316
Other and unspecified symptoms and signs involving cognitive functions and awareness	1	0.2	317
Other complications of procedures, not elsewhere classified	1	0.2	318
Other lack of expected normal physiological development	7	1.5	325
Other postprocedural respiratory disorders	1	0.2	326
Other secondary pulmonary hypertension	1	0.2	327
Other specified conduction disorders	2	0.4	329
Other specified degenerative diseases of nervous system	1	0.2	330
Other specified disorders of kidney and ureter	2	0.4	332
Other specified general symptoms and signs	3	0.6	335
Pancreas, unspecified	3	0.6	338
Pancreatic duct	1	0.2	339
Parkinson's disease	11	2.3	350
Perforation of intestine (nontraumatic)	1	0.2	351

**Immediate/primary cause of death**	**n**	**%**	**Cumulative frequency**
Pneumonia, unspecified	8	1.7	359
Pneumonitis due to food and vomit	7	1.5	366
Pulmonary embolism without mention of acute cor pulmonale	4	0.8	370
Respiratory arrest	10	2.1	380
Respiratory failure, unspecified	24	5.1	404
Secondary malignant neoplasm of bone and bone marrow	2	0.4	406
Secondary malignant neoplasm of other specified sites	1	0.2	407
Senility	1	0.2	408
Septicemia, unspecified	16	3.4	424
Sequelae of fracture of femur	1	0.2	425
Sequelae of other and unspecified cerebrovascular diseases	1	0.2	426
Shock, unspecified	1	0.2	427
Small intestine, unspecified	1	0.2	428
Stomach, unspecified	1	0.2	429
Stroke, not specified as hemorrhage or infarction	8	1.7	437
Sudden cardiac death, so described	1	0.2	438
Unknown	4	0.8	442
Unspecified dementia	6	1.3	448
Unspecified protein-energy malnutrition	5	1.1	453
Unspecified renal failure	5	1.1	458
Unspecified severe protein-energy malnutrition	7	1.5	465
Urinary tract infection, site not specified	1	0.2	466
Vascular dementia, unspecified	6	1.3	472
Vascular disorder of intestine, unspecified	1	0.2	473

**Immediate/primary cause of death**	**n**	**%**	**Cumulative frequency**
Ventricular tachycardia	1	0.2	474
